# Is Academic Achievement Related to Mediterranean Diet, Substance Use and Social-Cognitive Factors: Findings from Lebanese Adolescents

**DOI:** 10.3390/nu12051535

**Published:** 2020-05-25

**Authors:** Joyce Hayek, Francine Schneider, Maya Tueni, Hein de Vries

**Affiliations:** 1 School for Public Health and Primary Care (CAPHRI), Department of Health Promotion, Maastricht University, POB 616 6200 MD Maastricht, The Netherlands; francine.schneider@maastrichtuniversity.nl (F.S.); hein.devries@maastrichtuniversity.nl (H.d.V.); 2 Department of Biology, Nutrition and Dietetics, Faculty of Sciences II, Lebanese University, POB 90656 Fanar, Lebanon; mayatueni@hotmail.com

**Keywords:** health behaviors, Mediterranean diet, socio-cognitive factors, academic achievement, adolescents, Lebanon

## Abstract

There is substantial evidence that good academic performance significantly enhances the prospects of success for adolescents in terms of employment, social status, quality of life and health. Identifying which factors are correlated to good academic achievement and which factors may need to be addressed by policies is crucial. Despite its importance, there is insufficient data concerning factors associated with academic achievement in the Middle East, particularly Lebanon. This study assessed the association of lifestyle, socio-demographics and motivational factors with academic achievement of Lebanese adolescents. Grade 10 and 11 Lebanese adolescents aged 15 to 18 years (n = 600), from private and public schools in Beirut and the Mount Lebanon area, completed a multi-component questionnaire assessing health behaviors, socio-demographic characteristics and motivational factors. Height and weight were physically measured and, subsequently, Body Mass Index was calculated. Academic achievement was assessed using self-reported grades and was categorized into high and low. Associations between all factors and academic achievement were tested using logistic regression models. Adherence to the Mediterranean diet, high self-efficacy and intention were positively associated with academic achievement, whereas smoking was associated with poor achievement. Our findings support the need for targeting adolescents with an unhealthier lifestyle and focusing on socio-cognitive determinants interventions aimed at enhancing academic achievement.

## 1. Introduction

Academic performance is a subject of great importance to adolescents, parents, educators and researchers. Academically successful adolescents are believed to have better chances to be employed and earn better salaries [1]. In turn, higher income and social status were found to be linked to better health, for they allow privileged access to health care and health information, which helps individuals to better understand their health situation and therefore seek proper health services [2,3]. This is also true in the Lebanese context, as employment and higher income allows individuals to afford health insurance and access to quality health services which is not available for the unemployed or poor in the absence of universal health coverage [4]. Good academic performance is thus an essential precondition fostering the chances for later life in terms of employment and good health [5]. Yet, not all adolescents have the same opportunity for achieving good academic performances.

In Lebanon, academic performance has been found to range significantly; results from the Programme for International Student Assessment (PISA) measuring outcomes for math, science and reading showed that Lebanon performed significantly lower than the international average, and that Lebanese students are approximately four years of school behind other countries [6]. Lebanon’s low performance could result in significant differences concerning the future prospects of adolescents concerning employment, social status, quality of life and health. It is therefore important to study which factors are related to academic performance, and which actions could be undertaken at the national level, in order to potentially reduce this academic divide.

Academic performance is associated with several different factors, such as socio-demographics, lifestyle factors and motivational factors. Concerning socio-demographic factors, research suggests a relationship between socio-economic status and adolescents’ academic performance [7,8,9].

Adolescents from low socioeconomic status families tend to have poor academic performance, and are more likely to drop out of school [10,11]. This can be also influenced by living in poor neighborhoods and attending low quality and low-resourced schools and having restrained family resources [12,13]. Adolescents from a low socio-economic status (SES) are more likely to have parents with low levels of education [14] which can both negatively influence support from parents or setting academic performance goals [15,16]. Educated parents are more involved in their children’s education; like assisting their children in their homework and school activities, leading to a higher academic outcome [17,18]. 

Another significant factor affecting academic performance concerns health-related behaviors [19]. Poor nutrition has been found to negatively affect cognition; undernourished children suffer from impaired intellectual functioning, are less responsive and have difficulty concentrating [20]. This could be due to the role of some essential nutrients on brain function and cognitive capacity [21]. For instance, an insufficient intake of certain nutrients, such as iron and zinc, was found to be associated with short attention span and memory deficits [22,23]. Conversely, having a good quality diet was associated with higher academic scores [24,25,26]. Healthy eating habits such as breakfast consumption, higher fruit and vegetable intake and low intake of junk food have been associated with greater attention, better learning ability and higher academic achievement [27,28]. One of the dietary patterns that models healthy behaviors is the Mediterranean diet [29]. This dietary pattern is predominantly plant-based and rich in healthy fats and antioxidants [30]. Recent studies have linked greater adherence to the Mediterranean diet with higher academic achievement [31,32,33,34]. 

Academic achievement can also be positively associated with physical activity (PA). Although the evidence is not conclusive, studies showed that regular engagement in PA is associated with improved cognitive function, related to better attention, information processing and executive function [35,36]. Studies conducted on substance use and academic achievement indicated an inverse relationship; alcohol use and smoking are both associated with lower academic performance, and reciprocally low academic achievers are more likely to drink and smoke [37,38]. 

Socio-cognitive factors have been also linked to academic performance [39]. Adolescents who have strong beliefs in their academic capabilities were more motivated, worked harder and consequently performed better, compared to students with low self-efficacy [40,41,42]. In this study, we used the Integrated Change Model [43] by studying the role of attitudes, social norms, self-efficacy and intentions, as this model also posits influences of the distal variables described above. The I-Change Model integrates various social cognitive factors from various theories, such as Ajzen’s Theory of Planned Behavior [44], Bandura’s Social Cognitive Theory [45], the Transtheoretical Model [46], as well as the Health Belief Model [47]. The I-Change Model assumes, as many socio-cognitive models, that socio-cognitive factors and intention are important predictors of intentional behavior, as well as identifies factors determining these cognitions, such as the social cultural context, personal characteristics and engagement in other behaviors [43]. 

In conclusion, adolescents’ academic performance is a multifactorial process determined by various factors and these factors differ from country to country [25,48,49,50]. Several studies assessed the importance of these factors, but most studies did not use a comprehensive model which allows correction for multicollinearity between factors. Very little evidence is available supporting the evidence of these factors for Arab countries in general and for Lebanon in particular [51,52,53]. In addition, the gap between Lebanon and the international average point to a learning crisis [6], and highlights the need to obtain an overall description of the most important factors associated with academic performance of Lebanese adolescents in order to identify which factors are related to academic achievements and a future academic divide. 

Hence, the goal of this paper is to examine the relationship between academic performance of Lebanese adolescents with factors related to health behaviors, socio-demographics and motivational factors. This study will allow us to better understand which factors are associated with academic achievement of Lebanese adolescents in order to pave the way for evidence-based interventions that are culture based, as well as policies aiming at improving academic achievement on the long run.

## 2. Materials and Methods 

### 2.1. Study Design and Participants 

This was a descriptive study, with a cross-sectional design enrolling high school adolescents aged 15–18 years old, from grade 10 and 11, from private and public schools across Beirut and the Mount Lebanon area. These two regions were selected as they concentrate the majority of the Lebanese population, including approximately half of the Lebanese students, and are representative of the various religious and socio-demographic societies in Lebanon [54]. Ten schools (five private and five public) were randomly selected from the Ministry of Education’s list of schools in Beirut and the Mount Lebanon area following a stratified sampling design, the strata being public and private schools. Five private schools and five public schools were randomly selected out of the total number of schools in Beirut and the Mount Lebanon area, using the random sample of cases option in SPSS. The directors of these schools were approached face-to-face and provided with the study questionnaire, along with the objective of the study and seven (four private and three public) agreed to participate in the study. From these schools, all students from grade 10 and 11 were invited to participate in the survey. The Lebanese Ministry of Education and Higher Education reviewed and approved the study questionnaire (10/684; date: 1 March 2017). This study followed the ethical guidelines laid down in the Declaration of Helsinki [55], and ethics approval was obtained from the Scientific Committee of the Lebanese University. Written informed consent was obtained from all students and their parents prior to participating in the study. All students who were approached agreed to partake in the study, resulting in a total sample of 600 adolescents. 

### 2.2. Procedure

Trained dieticians visited the participating schools between March and April 2017, during school hours, to administer the questionnaire. All participating students completed the questionnaire by hand independently during class. The trained dieticians read aloud each question and the corresponding answers, and were present for any clarification. The questionnaire was completed by participants within approximately one hour. The trained dieticians also measured the heights and weights of participating students using standardized procedures [56] and calibrated equipment. Height was measured to the nearest 0.5 cm, using a portable stadiometer (ADE stadiometer, Germany), without shoes. Weight was measured to the nearest 0.1 kg, using a Secacalibrated electronic weighing scale (Hamburg, Germany) in light indoor clothes and barefoot. BMI was calculated as weight in kilograms divided by the square of height in meters (kg/m^2^). Overweight and obesity were defined according to cut-off values from the International Obesity Task Force for BMI of children aged 2–18 years, where centile curves were drawn, which, at age 18 years, passed through the widely used cut-off points of 30 and 25 kg/m^2^ for adult obesity and overweight [57].

### 2.3. Questionnaire

The questionnaire originally developed in English was translated in Arabic by a translator and then back translated to English by a native English translator [58] (see Supplementary Materials).

#### 2.3.1. Socio-Demographics

Socio-demographic questions included information on students’ sex (1 = male; 2 = female), age (1 = 15; 2 = 16; 3 = 17; 4 = 18), type of school (1 = public; 2 = private), educational level of parents (low = never went to school & primary school; medium = complementary & secondary school; high = technical school & university), working status of the parents (1 = working; 2 = not working), household crowding index, house ownership (1 = rented, 2 = privately owned), possession of a personal phone (1 = no; 2 = yes), having an internet connection (1 = no; 2 = yes), family structure (1 = living with both parents; 2 = other arrangements) and religion (1 = Christian; 2 = Muslim; 3 = atheist).

#### 2.3.2. Health Behaviors

##### Diet Quality

Dietary intake was assessed using a semi-quantitative Food Frequency Questionnaire (FFQ), adapted from a previous questionnaire that has been used among Lebanese children [59]. The FFQ measured the food intake over the past year. It includes 64 food and beverages commonly consumed in Lebanon and categorized into 10 food groups: breads and cereals, potatoes, rice and pasta, dairy products, fruits and juices, vegetables and salads, meats and alternatives, fats and oils, sweets and desserts, fast food and beverages. For each food item listed, a standard portion size was indicated. Students were asked to record the frequency of consumption either per day, per week, per month, per year or never. Students had the choice to report their intake either in reference portion size or in grams. Dietary habits were assessed with questions inquiring about regular intake of meals, breakfast consumption, snacking and frequency of eating out. 

Data from the FFQ and dietary habits questions were used to calculate the KIDMED index (Mediterranean Quality Index for children and adolescents) [60]. The KIDMED index measures the degree of adherence to the Mediterranean diet (MeD) by measuring the consumption of 16 items, of which 12 are positively scored and four negatively scored. Items denoting a positive association to the MeD are assigned a value of +1: (1) fruit/fruit juice every day, (2) second fruit every day, (3) vegetables regularly once a day, (4) vegetables more than once a day, (5) fish at least 2–3 times/week, (6) pulses more than once a week, (7) pasta or rice consumption ≥5/week, (8) cereals or grains for breakfast, (9) nuts at least 2–3 times/week, (10) regular use of olive oil, (11) a dairy product for breakfast and (12) two yoghurts and/or some cheese (40 g) daily. 

Items denoting a negative association to the MeD are assigned a value of −1: (1) fast food >1/week, (2) skipping breakfast, (3) commercially baked goods or pastries for breakfast, (4) taking sweets and candy several times every day. 

The total score ranges from 0 to 12, with higher scores indicating higher adherence to the MeD [61]. 

##### Smoking and Alcohol

Smoking status was assessed by two questions: ’Have you ever smoked 100 cigarettes in your life?’ (yes or no) and ’During the past 30 days, on how many days did you smoke cigarettes?’ [62]. Participants were categorized as (1) never smokers (those who had not smoked 100 cigarettes in their lifetime and had not smoked in the last 30 days), (2) former smokers (those who had smoked 100 cigarettes in their lifetime but had not smoked in the last 30 days), (3) current smokers (those who had smoked 100 cigarettes in their lifetime and had smoked in the last 30 days) [63]. 

Prevalence of alcohol consumption in the past 30 days was assessed with the question: ‘During the past month, on how many days did you drink alcohol?’ the responses were ‘0 days; 1 or 2 days; 3 to 5 days; 6 to 9 days; 10 to 19 days; 20 to 29 days; All 30 days’. In line with the categorization used in the Global School Health Survey, the responses were then dichotomized into (1) no = 0 days and (2) yes = 1–30 days [62].

##### Breakfast Intake

The breakfast questions inquired about breakfast intake and frequency. Breakfast consumers were defined as adolescents who consumed any food from at least one food group, and within three hours of waking up [64]. Frequency of intake was assessed with the question: ‘How many days of the week do you eat breakfast?’, and was categorized as: (1) rare (0–2 days/week), (2) occasional (3–4 days/week) and (3) frequent (5–7 days/week). 

##### Physical Activity

Physical activity was assessed using the short version of the International physical activity questionnaire (IPAQ). The IPAQ has been shown to be a reliable and valid tool to obtain estimates of PA [65,66,67]. The questionnaire asks about three specific levels of activity: walking, moderate and vigorous-intensity activities and their frequency (days per week) and duration (minutes per day). Total PA was calculated by multiplying time spent in each activity intensity by its estimated metabolic equivalent METs estimated at 3.3 for walking, 4.0 for moderate intensity activity and 8.0 for vigorous intensity activity (e.g., walking MET-minutes/week = 3.3 x walking minutes x walking days). MET-minutes/week for each activity are summed to derive the total PA MET-minutes/week [68]. Three categories of PA were assigned on the basis of MET-min/week: (1) low: <600, (2) moderate: at least 600 and (3) high: at least 3000. 

#### 2.3.3. Socio-Cognitive Factors

The questionnaire was based on the I-Change Model addressing socio-demographic, cultural, ecological and motivational factors [43]. The I-Change Model has been used to assess a variety of health behaviors, including nutrition behavior [69,70]. 

Attitude was measured by using the responses to four questions addressing the pros and cons of getting good academic grades. Two questions measured positive attitudes towards getting good grades (‘Getting good academic grades is a good help for getting a good job/will get me compliment from my parents’). The responses were coded from −2 to +2 (−2 = strongly disagree to +2 = strongly agree). Two questions measured the negative attitude towards getting good grades (‘Getting good academic grades means that I have to work too hard/will cause disapproval among my friends’). For the negative statement, responses were reverse coded from +2 to −2 (2 = strongly disagree to −2 = strongly agree), so that higher scores indicate a more positive attitude toward getting good academic grades. Social norms were measured using the responses to three questions asking if important people in their environment (both parents and teachers) expect them to get good academic grades (‘My father/my mother/my teacher expects me to get good academic grades’) on a five-point Likert scale (+2 = strongly agree, −2 = strongly disagree).

Adolescents’ academic self-efficacy was measured using five question scale (‘I find it easy to get good academic grades/to concentrate at school for getting good academic grades/to master the skills that are taught in class this year/to concentrate on school work when I am at home/to finish all my school work’). All the questions were measured on a five-point Likert scale (+2 = strongly agree, −2 = strongly disagree), with higher scores indicating higher self-efficacy. A mean score for self-efficacy was composed (α = 0.76).

Intention to get good academic grades was assessed by one question: ‘I intend to get good academic grades’ on a five-point Likert scale (+2 = strongly agree, −2 = strongly disagree) [71,72]. 

#### 2.3.4. Main Outcome Measure

Academic achievement was measured using the student’s general average, which is the result of the performance of the student in all school subjects during a specific semester. The general average is the standard instrument for the assessment of the academic achievement of students in Lebanese schools. Most schools use a 0–20 scale, where the passing grade is 10 out of 20. This means that if students score lower than 10 out of 20, they fail their academic semester, whereas if they score 10 or above they pass. Students were asked to report their general average of the last semester. Academic achievement was dichotomized into (1) high ≥ 10 and (0) low < 10. 

### 2.4. Statistical Analysis

Data entry and analysis were performed on SPSS statistical software, version 21. (SPSS Inc. Chicago, IL, USA) and a *p* value < 0.05 was considered to be significant. 

Data cleaning was performed on a sample of 50 questionnaires that were completely checked for errors. The error rate was lower than 1%; thus, data entry was considered adequate. Missing data were not replaced for this analysis, due to their low percentage (<10%) [73].

A descriptive analysis was performed using means and standard deviations for all continuous variables, whereas numbers and percentages were used for categorical variables.

The Pearson Chi-square was used to examine the associations between categorical variables and both categories of academic achievement. Fisher’s exact test was also used when the expected frequency was less than 5. Independent Samples T-test was used to evaluate the differences between the means of continuous measures for both categories of academic achievement. 

A multivariate logistic regression (high academic achievement = 1/low academic achievement = 0) was carried out to identify which factors were independently associated with academic achievement using the Enter method. Variables which obtained *p* < 0.2 in the bivariate analysis were entered in the model [74,75]. Independent variables were introduced by blocks: Model 1 included socio-demographic and school variables. Model 2 also contained BMI classes and health behaviors variables, and in Model 3, the socio-cognitive variables were added. Adjusted Odds ratios (aOR) were presented in tables with 95% confidence intervals (CI). We evaluated the models using Nagelkerke R^2^, the Omnibus test, Hosmer-Lemeshow goodness-of-fit tests and the percentage of correctly classified cases [76]. 

## 3. Results

### 3.1. Description of the Sample

Out of the 600 distributed questionnaires, 563 (94%) were used for data analysis and 36 (6%) were removed for being almost empty or incomplete. The final sample consisted of 50.1% male and 49.9% female participants, with a mean age of 15.8. Out of the study participants, 66% were in grade 10 and 34% in grade 11, while 62.5% of subjects attended private school and 37.5% attended public school. The proportion of parents with high educational level was of 52.5% for fathers and 55.1% for mothers. In all, 95.1% of fathers and 47.1% of mothers were working. The average BMI for the subjects in the study was 23.67 (SD = 4.48), and 39.2% of the participants were overweight/obese. Out of the 563 participants, 80.6% had an average grade of 10 or above and 19.4% scored lower than 10 (Table 1).

### 3.2. Socio-Demographics and Academic Achievement

The prevalence of high academic achievement was significantly greater among females compared to males (*p* = 0.026). High academic achievement was also more prevalent in private school students (*p* < 0.001), adolescents living with both parents (*p* = 0.042) and adolescents with normal weight (*p* = 0.049). No significant effects were found for factors such as parents’ education, working status and religion (Table 1). 

### 3.3. Health Behavior and Academic Achievement

Adherence to the MeD was particularly low (3.77 ± 2.51). Diet quality assessed by adherence to the MeD was significantly correlated with academic achievement (Table 2): the higher the adherence to the MeD, the higher the probability to have high academic achievement (*p* < 0.001). Additionally, the prevalence of high academic achievement was significantly greater among adolescents with medium levels of PA compared to low levels (*p* < 0.001). Non-smokers and past smokers were also significantly more likely to have high academic achievement compared to current smokers (*p* = 0.002) (Table 2). No significant associations of academic achievement with snacking frequency and type, alcohol consumption and breakfast intake were found.

### 3.4. Socio-Cognitive Factors and Academic Achievement

The prevalence of having high academic achievement was highest among students who reported the highest level of social norms towards getting good grades from their teacher, compared to their parents (*p* = 0.028). Moreover, students with greater reported academic self-efficacy towards being able to achieve academic performances and with stronger intentions toward getting good grades were more likely to have high academic achievement (*p* < 0.001) (Table 3). No significant associations of academic achievement and attitude were found.

### 3.5. Multivariate Analysis

Three multivariate regressions are shown in Table 4. The first model including all socio-demographic factors revealed that adolescents from private schools and girls were more likely to have high academic achievements. In the second model, adding health behaviors to Model 1, type of school and gender were no longer significant. The odds of having high academic achievement were significantly lower for overweight and obese adolescents (aOR: 0.52; 95% CI 0.28–0.95), as well as for current smokers (aOR: 0.29; 95% CI 0.13–0.67). Adolescents with medium levels of PA were 2.73 times more likely to have high academic achievement compared to low level groups. Finally, the odds of having high academic achievement were significantly greater with higher adherence to the MeD (aOR: 1.39; 95% CI 1.21–1.59). In the third and final model, in which socio-cognitive factors were also added, the odds of having high academic achievement remained significantly greater for students with high adherence to the MeD (aOR:1.34; 95% CI 1.15–1.56). Similarly, current smokers remained less likely to have high academic achievement compared to those who do not smoke (aOR: 0.38; 95% CI 0.15–0.93), but PA and BMI were no longer significant. Self-efficacy seemed to have the most prominent effect (aOR: 1.81; 95% CI 1.15–2.84) followed by intention (aOR: 1.40; 95% CI 1.01–1.95).

Variables with a *p*-value < 0.2 in the bivariate analysis were included in the multivariate analysis, to make sure that all pertinent and potentially predictive variables are studied.

**Model 1**: Variables entered: Type of school, Gender, Mother’s educational level, Family structure, Religion. Omnibus test *p*-value < 0.001/Hosmer-Lemeshow test *p*-value = 0.341.
  Nagelkerke R^2^ = 0.167/Overall predicted percentage = 81.9%.
**Model 2**: Variables entered: Variables in Model 1 + BMI classes, Regular meal pattern, Smoking status, Prevalence of Alcohol consumption in the past 30 days, Habitual breakfast consumption, PA level/ TOTAL MET_MIN/WEEK, KIDMED Index Adherence to the MeD Diet. Omnibus test *p*-value < 0.001/Hosmer-Lemeshow test *p*-value = 0.599. Nagelkerke R^2^ = 0.360/Overall predicted percentage = 85.1%.
**Model 3**: Variables entered: Variables in Model 2 + Getting good grades means that I have to work too hard, Getting good grades means will cause disapproval among my friends, My teacher expects that I get good academic grades, Self-efficacy Total, Intention.
  Omnibus test *p*-value < 0.001/Hosmer-Lemeshow test *p*-value = 0.760. Nagelkerke R^2^ = 0.429/Overall predicted percentage = 88.0%.

## 4. Discussion

Even though, several studies have been conducted to examine the factors associated with academic performance of adolescents [24,48,77], very few assess the factors in one comprehensive model, and may not correct for overlap between potential factors. The current study examined the relationship between academic achievement of Lebanese adolescents with health behaviors, socio-demographics and motivational factors in order to identify modifiable factors to foster future academic achievements in this group of adolescents.

### 4.1. Socio-Demographics and Academic Achievement

In the bivariate analysis, and when entered in the multivariate model alone, gender and type of school were significantly associated with achievement. Academic achievement was significantly higher in girls and adolescents from private schools. The result that girls outperformed boys is a common finding [77,78], and has been explained by several theories, among which are the differences between girls and boys in interests and attitudes towards learning [79]. Regarding the type of school, in Lebanon, the two sectors public and private are an indicator of the different socio-economic backgrounds. Private schools have high tuition fees and, thus, are more likely to attract adolescents from higher SES, whereas public schools are practically free of charge and usually adolescents enrolled in the public sector come from low SES families. Our finding that adolescents from private schools were found to have higher achievement is in line with previous studies [80], and might be explained by the fact that adolescents enrolled in private schools benefit from cultural capital and material resources leading to higher performance [81]. However, gender and type of school were overshadowed when health behaviors were added to the model, and became insignificant. 

### 4.2. Health Behavior and Academic Achievement

Our results extend the findings of previous research, and demonstrate a significant association between diet quality and academic achievement [34,82,83]. A higher adherence to the MeD was positively associated with high academic achievement. Our observations are in agreement with prior research, where high adherence to the MeD was related to critical thinking, greater capacity for effort [31], higher academic performance, and the higher the adherence, the better the academic scores [32,33]. It is notable that, in our study, this finding was also found after correcting for parental SES, thus suggesting an independent effect of MeD. The MeD is a healthy eating pattern characterized by high intakes of plant food, olive oil, fish and limited intake of meat, dairies and sweets [84]. The positive association between MeD and academic achievement could be related to the richness of this diet in key nutrients, with antioxidant and anti-inflammatory properties which were found to positively influence cognitive function [85,86,87]. High adherence to the MeD is associated with a higher intake of antioxidant rich foods, such as fruits and vegetables and phytochemicals, particularly polyphenols [88], which were found to reduce inflammation and oxidative stress thus leading to better cognitive performance [88,89]. Other predominant nutrients in the MeD are omega-3 fatty acids, known for their neuroprotective properties and importance in brain development and function [87,88]. On another note, and beyond the effect of distinct dietary components, the MeD diet is considered an overall healthy and balanced diet [90]. A healthy diet was found to positively relate to better mental well-being, self-esteem, lower anxiety and stress [91,92] which, in turn, can improve cognition and performance. The latter implies that the promotion of MeD is worth considering for enhancing academic performances, as well as overall better health. It is important to note that adherence to MeD among our sample was mainly low (mean score 3.77); in fact, a recent study showed that Lebanese adolescents were mostly following a Western dietary pattern, characterized by high intakes of fast food and refined sweets [93], which has been found to negatively impact academic performance [94]. These findings highlight the need for further work on identifying the determinants of adherence to the MeD in order to preserve and promote this cultural healthy dietary pattern in Lebanon.

Furthermore, this study confirms existing evidence that substance use predicts poor educational achievement [37]. Our results indicate that adolescents who smoke were more likely to have low academic achievement. The association of smoking and poor performance is well established [95]; however, the underlying mechanisms remain unclear. What is recognized is that smoking and poor academic achievement mutually influence each other [38]. Smoking is associated with a higher likelihood of poor academic achievement, conversely, academic failure is associated, through psychosocial mediating factors (like favorable attitude towards smoking and weaker self-efficacy to refuse smoking), with a higher probability of smoking [96]. Consequently, efforts aimed at preventing the onset of smoking among adolescents in Lebanon should be pursued, as they not only foster good physical health but also cognitive health.

### 4.3. Socio-Cognitive Factors and Academic Achievement

With regards to socio-cognitive factors, our results show that having a higher academic self-efficacy and stronger intention towards getting good grades are positively associated with higher academic achievement. These findings are supported by the past literature, indicating that the higher the self-efficacy, the better the academic performance [97,98]. In fact, self-efficacy and intentions are linked [99]; individuals with high self-efficacy are more likely to set higher goals and develop a stronger intention to achieve these goals [100], in this case, getting good academic grades. Personal beliefs about efficacy can be stronger predictors of academic achievement then actual abilities [101]; students often have poor academic achievement not because they are incompetent, but rather due to not believing they have the capabilities to succeed [102]. Highly efficacious students are highly motivated, work harder and more persistently towards achieving academic tasks, and consequently perform better than students with lower efficacy beliefs [103]. The literature also suggests a reciprocal relation between self-efficacy and academic performance; past academic success enhances students ‘efficacy beliefs, while experiencing failure lowers it [104]. Mastery experience is indeed one of the most influential factors affecting self-efficacy but not the only source. Vicarious experience, verbal persuasion and physiological reactions can also foster self-efficacy [102,105]. Sources of self-efficacy can differ across culture [106]; consequently, future research is needed to investigate which factor has the greatest impact on self-efficacy of Lebanese adolescents, so as to best realize positive efficacy beliefs and consequently intentions toward achieving academic grades. 

Although borderline significant, our results suggest that negative attitudes towards academic achievements and disapproval by friends may play a role. Consequently, our findings underline the need for a more in-depth research towards the role of these socio-cognitive factors, and how to change them.

### 4.4. Strength and Limitations

Very few studies have done this kind of research in youth from developing countries [24,52,53] and evidence from Lebanon is much needed. To the best of our knowledge, this is the first study to examine the association of academic achievement with health behaviors, socio-demographics and socio-cognitive factors among Lebanese adolescents. The strengths of this descriptive study also include the comprehensive model used comprising of a wide range of factors and the objective method to measure weight and height. More studies in Arab cultures are needed, to identify whether similar patterns can also be observed in related countries. One limitation of this study was that grades were self-reported and the possibility of students overestimating their academic performance should be considered. However, previous research indicates that self-reported grades can be a reliable measure of academic performance, since they are comparable to academic transcripts [26,107]. Furthermore, our results showing that the majority of students (80.6%) had a high achievement level, scoring higher than 10 and passing the school semester, is comparable to those of the Center for Educational Research and Development, in which 83.3% are succeeding vs. 16.7% who are failing [108]. 

In addition, our study had a cross-sectional design, allowing us to test associations rather than infer causal relationships, further longitudinal studies recruiting schools to engage in research for several measurements is needed to confirm the associations. Finally, the majority of our sample comes from private schools (62.5% vs. 37.5% from public schools), which is comparable with statistics showing that private schools in Lebanon account for the majority of total enrollment [108]. However, caution should be exerted in generalizing the results to the whole adolescents’ population in Lebanon. The sample population was selected from Beirut, the capital, and Mount Lebanon, these two areas have the highest concentration of people and are representative of the various religious and socio-demographic societies in Lebanon. While the distribution of the study sample by sex and school sector was similar to that of the Lebanese secondary student population [108], the sample is not at a national level and, consequently, this limit the generalizability of the results. 

## 5. Conclusions and Implications

Our findings of an association between diet, smoking and academic achievement adds to the long-existing evidence on the relation of health to academic success, and provides further rationale on the importance of promoting healthy lifestyle habits among youth. Most importantly, this study shows a sub-optimal level of adherence to the MeD (mean score 3.77). Lebanese adolescents are moving away from this traditional healthy dietary pattern towards a more Westernized diet. The latter highlights the need to raise awareness among Lebanese youth on the benefits of the MeD and its importance for both physical and cognitive health.

At the school level, this can be done by incorporating nutrition education into the school curriculum, educating adolescents about the nutritional benefits of the MeD and encouraging greater adherence by minimizing the sale of low nutrient, high energy foods in school shops, and instead provide healthy alternatives to students. Nutrition sessions should also target parents, as they are key players in helping their children adopt healthy behaviors and maintain healthy habits in the home environment [109]. Health educators need to also tackle the subject of smoking, discuss the negative effect of tobacco use, the hazards of smoking, teach adolescents how to be aware of social influences and how to resist them. School programs are considered amongst the most effective strategies to reduce smoking prevalence in adolescents. 

By integrating health and nutrition education into the regular school curriculum, schools are not only improving students’ cognitive health and learning, but are also supporting adolescents’ long-term health and wellness as to chronic diseases prevention, healthy weight and long life-expectancy. Our results also show a high proportion of overweight and obesity (39.2%). Promoting healthy eating and participation in regular activity within schools can help adolescents acquire healthy habits and curb the progression of obesity.

Furthermore, given the strong association of self-efficacy with academic achievement, it is important to promote the development of students’ self-efficacy. Educators can foster students’ academic self-efficacy by providing frequent positive feedback, encouragement and guidance. Group activities can be also beneficial; observing peers succeeding will motivate them to try and do the same [110]. Parents can play a role too in nurturing their children’s self-efficacy by engaging in their academic activities, praising their efforts when deserved and showing recognition for a job well done, but also providing honest feedback when they fail and encouraging and challenging them to do better.

Lastly, to carry over and complement the efforts done at the school and home level, national policies and strategies addressing access to healthy food, physical activity and tobacco use need to be established. Local authorities have the power and responsibility to shape the environment into a healthy one and enable adolescents to make healthy choices. Community-based interventions and environmental support involving all sectors of society are recommended to facilitate sustainable healthy behavioral change.

## Figures and Tables

**Table 1:** Association of Socio-demographics and Anthropometric Measurements with Academic Achievement.

Variables	Total Number 563 (100%) N (%)	Low Academic Achievement 109 (19.4%) N (%)	High Academic Achievement 454 (80.6%) N (%)	Test Statistic (df)	*p*
**Type of school**				χ^2^ (1) = 12.663	<0.001 ^a^
- Public	211 (37.5%)	57 (27%)	154 (73.0%)		
- Private	352 (62.5%)	52 (14.8%)	300 (85.2%)		
**Gender**				χ^2^ (1) = 4.925	0.026 ^a^
- Boys	282 (50.1%)	65 (23%)	217 (77.0%)		
- Girls	281 (49.9%)	44 (15.7%)	237 (84.3%)		
**Age**				χ^2^ (3) = 0.270	0.966 ^a^
-15	234 (41.6%)	44 (18.8%)	190 (81.2%)		
-16	225 (40%)	43 (19.1%)	182 (80.9%)		
-17 -18	85 (15.1%) 19 (3.4%)	18 (21.2%) 4 (21.1%)	67 (78.8%) 15 (78.9%)		
**Crowding index**				χ^2^ (1) = 1.201	0.273 ^a^
- <1 person/room	182 (32.5%)	30 (16.5%)	152 (83.5%)		
- ≥1 person/room	378 (67.5%)	77 (20.4%)	301 (79.6%)		
**House ownership**				χ^2^ (1) = 0.360	0.549 ^a^
- Rented	108 (19.3%)	23 (21.3%)	85 (78.7%)		
- Privately owned	453 (80.7%)	85 (18.8%)	368 (81.2%)		
**Internet connection**				χ^2^ (1) = 1.592	0.207 ^a^
- No	29 (5.2%)	3 (10.3%)	26 (89.7%)		
- Yes	534 (94.8%)	106 (19.9%)	428 (80.1%)		
**Personal smart phone**				χ^2^ (1) = 0.253	0.778 ^c^
- No	20 (3.6%)	3 (15%)	17 (85.0%)		
- Yes	543 (96.4%)	106 (19.5%)	437 (80.5%)		
**Father’s educational level**				χ^2^ (2) = 0.471	0.790 ^a^
- Low (Illiterate & Primary school) - Moderate	31 (6.5%)	5 (16.1%)	26 (83.9%)		
(Complementary & Secondary school)	195 (41%)	38 (19.5%)	157 (80.5%)		
-High (Technical & University)	250 (52.5%)	43 (17.2%)	207 (82.8%)		
**Mother’s educational level**				χ^2^ (2) = 4.529	0.104 ^a^
- Low (Illiterate & Primary school)	22 (4.4%)	6 (27.3%)	16 (72.7%)		
- Moderate (Complementary & Secondary school)	204 (40.6%)	43 (21.1%)	161 (78.9%)		
-High (Technical & University)	277 (55.1%)	41 (14.8%)	236 (85.2%)		
**Father work**				χ^2^ (1) = 0.326	0.568 ^a^
- Not working	27 (4.9%)	4 (14.8%)	23 (85.2%)		
- Working	525 (95.1%)	101 (19.2%)	424 (80.8%)		
**Mother work**				χ^2^ (1) = 0.088	0.767 ^a^
- Not working	296 (52.9%)	59 (19.9%)	237 (80.1%)		
- Working	264 (47.1%)	50 (18.9%)	214 (81.1%)		
**Family structure**				χ^2^ (1) = 4.140	0.042 ^a^
- Live with both parents	507 (90.4%)	92 (18.1%)	415 (81.9%)		
- Other arrangements	54 (9.6%)	16 (29.6%)	38 (70.4%)		
**Religion**				χ^2^ (3) = 5.552	0.115 ^c^
- Christian	434 (78.1%)	77 (17.7%)	357 (82.3%)		
- Muslim	110 (19.8%)	27 (24.5%)	83 (75.5%)		
- Atheist	10 (1.8%)	4 (40%)	6 (60%)		
- Druze	2 (0.4%)	0 (0%)	2 (100%)		
**Weight (km)**	66.44 ± 15.50	69.67 ± 16.00	65.65 ± 15.30	t (559) = 2.440	0.015 ^b^
**Height (cm)**	167.12 ± 8.85	168.51 ± 8.61	166.77 ± 8.89	t (561) = 1.845	0.066 ^b^
**BMI (kg/m^2^)**				χ^2^ (2) = 6.013	0.049 ^a^
-Underweight	25 (4.5%)	6 (24.0%)	19 (76.0%)		
- Normal	316 (56.3%)	50 (15.8%)	266 (84.2%)		
- Overweight/Obese	220 (39.2%)	53 (24.1%)	167 (75.9%)		

Notes: ^a^ *p*-value for the chi-square test, ^b^ *p*-value for the Independent Samples T-test, ^c^ *p*-value for Fisher’s exact test.

**Table 2:** Association between Health Behaviors and Academic Achievement.

Variables	Total Number N (%)	Low Academic Achievement	High Academic Achievement	Test Statistic (df)	*p*
**Regular meal pattern**				χ^2^ (1) = 2.314	0.128 ^a^
- No	406 (72.1%)	85 (20.9%)	321 (79.1%)		
- Yes	157 (27.9%)	24 (15.3%)	133 (84.7%)		
**Snacking frequency per day**				χ^2^ (3) = 2.283	0.516 ^a^
- No	36 (6.4%)	8 (22.2%)	28 (77.8%)		
-Once	153 (27.2%)	34 (22.2%)	119 (77.8%)		
-Twice	236 (41.9%)	39 (16.5%)	197 (83.5%)		
-3 times or more	138 (24.5%)	28 (20.3%)	110 (79.7%)		
**Type of snack**				χ^2^ (2) = 1.763	0.414 ^a^
-Sandwich	76 (14.4%)	17 (22.4%)	59 (77.6%)		
-Fruits & Vegetables	124 (23.4%)	19 (15.3%)	105 (84.7%)		
-Sweets, Candies & Salty crackers	329 (62.2%)	65 (19.8%)	264 (80.2%)		
**Smoking status**				χ^2^ (2) = 11.851	0.002 ^b^
-Never	505 (90.3%)	89 (17.6%)	416 (82.4%)		
-Past	6 (1.1%)	1 (16.7%)	5 (83.3%)		
-Current	48 (8.6%)	19 (39.6%)	29 (60.4%)		
**Do you drink alcohol**				χ^2^ (1) = 1.513	0.219 ^a^
- No	229 (40.7%)	50 (21.8%)	179 (78.2%)		
- Yes	334 (59.3%)	59 (17.7%)	275 (82.3%)		
**Prevalence of Alcohol consumption in the past 30 days**				χ^2^ (1) = 2.019	0.155 ^a^
- No	255 (45.3%)	56 (22%)	199 (78.0%)		
- Yes	308 (54.7%)	53 (17.2%)	255 (82.8%)		
**Sleeping hours**				χ^2^ (1) = 0.239	0.625 ^a^
-<8 h	371 (65.9%)	74 (19.9%)	297 (80.1%)		
-≥8 h	192 (34.1%)	35 (18.2%)	157 (81.8%)		
**KIDMED Index Adherence to MeD**	3.77 ± 2.51	2.30 ± 2.07	4.11 ± 2.46	t (561) = −7.090	<0.001 ^c^
**Breakfast intake**				χ^2^ (1) = 1.053	0.305 ^a^
- No	139 (25.6%)	31 (22.3%)	108 (77.7%)		
- Yes	404 (74.4%)	74 (18.3%)	330 (81.7%)		
**Habitual breakfast consumption**				χ^2^ (2) = 5.338	0.069 ^a^
-Rare (0–2 days)	48 (8.5%)	10 (20.8%)	38 (79.2%)		
-Occasional (3–4 days)	135 (24.0%)	35 (25.9%)	100 (74.1%)		
-Frequent (5–7 days)	380 (67.5%)	64 (16.8%)	316 (83.2%)		
**PA level**				χ^2^ (2) = 15.834	<0.001 ^a^
- Low	184 (32.8%)	39 (21.2%)	145 (78.8%)		
- Medium	155(27.6%)	14 (9.0%)	141 (91.0%)		
- High	222 (39.6%)	56 (25.2%)	166 (74.8%)		

Notes: ^a^ *p*-value for the chi-square test, ^b^ *p*-value for Fisher’s exact test, ^c^ *p*-value for the Independent Samples T-test.

**Table 3:** Association of Socio-cognitive Factors and Academic Achievement.

	Total Number	Low Academic Achievement	High Academic Achievement	Test Statistic (df)	*p* ^a^
**Getting good grades is a good help for getting a good job**	0.87 ± 0.98	0.97 ± 0.94	0.85 ± 0.98	t (559) = 1.213	0.226
**Getting good grades will get me compliment from my parents**	1.15 ± 0.89	1.13 ± 0.90	1.15 ± 0.89	t (558) = −0.194	0.846
**Getting good grades means that I have to work too hard**	−0.68 ± 0.96	−0.83 ± 1.00	−0.65 ± 0.94	t (561) = −1.710	0.088
**Getting good grades means will cause disapproval among my friends**	1.05 ± 1.05	0.88 ± 1.14	1.09 ± 1.03	t (561) = −1.831	0.068
**My father expects that I get good academic grades**	1.03 ± 0.98	1.11 ± 0.99	1.02 ± 0.97	t (556) = 0.923	0.357
**My mother expects that I get good academic grades**	1.15 ± 0.91	1.24 ± 0.80	1.13 ± 0.94	t (559) = 1.176	0.240
**My teacher expects that I get good academic grades**	0.69 ± 0.90	0.48 ± 1.13	0.73 ± 0.83	t (135) = −2.216	0.028
**Self-efficacy Total**	0.25 ± 0.74	−0.17 ± 0.81	0.35 ± 0.68	t (140) = −6.002	<0.001
**Intention**	1.15 ± 0.90	0.75 ± 1.19	1.25 ± 0.80	t (132) = −4.116	<0.001

Notes: ^a^ *p*-value for the Independent Samples T-test.

**Table 4:** Association of Socio-demographics, Health Behaviors, Socio-cognitive Factors with Academic Achievement in Lebanese Adolescents.

	Model 1			Model 2			Model 3		
Variables	aOR	95% CI	*p*	aOR	95% CI	*p*	aOR	95% CI	*p*
**Type of school**									
- Public	1			1			1		
- Private	2.39	1.30–4.39	0.005	1.80	0.90–3.60	0.097	2.02	0.96–4.25	0.064
**Gender**									
- Boys	1			1			1		
- Girls	1.96	1.17–3.29	0.011	1.35	0.74–2.48	0.311	1.53	0.77–3.03	0.224
**Mother’s educational level**									
- High (Technical & University)	1			1			1		
- Low (Illiterate & Primary school)	0.68	0.21–2.21	0.523	0.50	0.13–1.93	0.317	0.64	0.16–2.58	0.533
- Moderate (Complementary & Secondary school)	0.82	0.47–1.45	0.501	0.72	0.38–1.36	0.309	0.74	0.37–1.45	0.375
**Family structure**									
- Other arrangements	1			1			1		
- Live with both parents	1.43	0.64–3.20	0.385	1.21	0.48–3.08	0.682	0.82	0.30–2.23	0.698
**Religion**									
- Christian	1			1			1		
- Muslim - Druze - Atheist	0.78	0.43–1.42	0.417	0.82	0.40–1.71	0.599	0.81	0.37–1.77	0.592
**BMI (kg/m^2^)**									
- Normal				1			1		
- Underweight				0.32	0.09–1.11	0.073	0.31	0.08–1.13	0.076
- Overweight/Obese				0.52	0.28–0.95	0.032	0.55	0.29–1.04	0.068
**Regular meal pattern**									
- No				1			1		
- Yes				0.70	0.34–1.45	0.341	0.63	0.29–1.36	0.236
**Smoking status**									
- Never				1			1		
- Past				2.52	0.12–52.28	0.550	2.23	0.12–41.42	0.592
- Current				0.29	0.13–0.67	0.004	0.38	0.15–0.93	0.034
**Prevalence of Alcohol consumption in the past 30 days**									
- No				1			1		
- Yes				1.44	0.75–2.77	0.272	1.31	0.65–2.62	0.446
**Habitual breakfast consumption**									
- Rare (0–2 days)				1			1		
- Occasional (3–4 days)				0.47	0.15–1.48	0.199	0.30	0.08–1.06	0.061
- Frequent (5–7 days)				0.55	0.18–1.67	0.290	0.38	0.11–1.29	0.121
**PA level**									
- Low				1			1		
- Medium				2.73	1.13–6.60	0.026	2.34	0.91–6.03	0.077
- High				0.68	0.36–1.30	0.243	0.59	0.29–1.18	0.132
**KIDMED Index Adherence to the Med Diet**				1.39	1.21–1.59	<0.001	1.34	1.15–1.56	<0.001
**Getting good grades means that I have to work too hard**							1.15	0.83–1.59	0.412
**Getting good grades means will cause disapproval among my friends**							1.03	0.76–1.39	0.866
**My teacher expects that I get good academic grades**							1.38	0.99–1.92	0.061
**Self-efficacy Total**							1.81	1.15–2.84	0.010
**Intention**							1.40	1.01–1.95	0.047

aOR = adjusted Odds Ratio; CI = confidence interval; BMI = Body Mass Index. Dependent variable: High/low Academic Achievement.

## Supplementary Materials

The following are available online at https://www.mdpi.com/2072-6643/12/5/1535/s1, Supplementary file describing factors measured and questions asked. Table S1: Socio-demographics, Table S2: Dietary behavior, Table S3: Breakfast Questions, Table S4: Physical Activity, Table S5: Socio-cognitive factors.
