# Supplementary material for: Is Academic Achievement Related to Mediterranean Diet, Substance Use and Social-Cognitive Factors: Findings from Lebanese Adolescents
Source: Nutrients. 2020 May 25;12(5):1535. doi: 10.3390/nu12051535 (PMC7284451; doi:10.3390/nu12051535)
Supplement: Supplementary file 1 [file nutrients-12-01535-s001.pdf]

Supplementary Table S1: Socio-demographics

**Socio-demographics**	
Questions	Answer
Gender	1=Male<br>2=Female
How old are you?	1=15 years old<br>2=16 years old<br>3=17 years old<br>4=18 years old
How many rooms are there in your house? (excluding bathroom, kitchen)	Specify in number:<br>___________________
How many people live with you in your home (excluding maids)?	Specify the number:<br>___________________
The house in which you are living is:	1=Rented<br>2=Privately owned
Do you have internet connection at your house?	1=No<br>2=Yes
Do you personally own a smart phone?	1=No<br>2=Yes
What is the highest level of education that your father has completed?	1=Never went to school<br>2=Completed primary school<br>3=Completed complementary school<br>4=Completed secondary school<br>5=Technical school<br>6=University<br>7=I don't know
What is the highest level of education that your mother has completed?	1=Never went to school<br>2=Completed primary school<br>3=Completed complementary school<br>4=Completed secondary school<br>5=Technical school<br>6=University<br>7=I don't know
How many days a week does your father work?	1=He doesn't work<br>2=1 day/week<br>3=2 days/week<br>4=3 days/week<br>5=4 days/week<br>6=5 or more days/week
How many days a week does your mother work?	1=She doesn't work<br>2=1 day/week<br>3=2 days/week<br>4=3 days/week<br>5=4 days/week<br>6=5 or more days/week
Who lives with you in your home?<br>Family structure	1=Both parents<br>2=Mother and not father<br>3=Father and not mother<br>Other, specify ________________

Supplementary Table S2: Dietary behavior

**Dietary behavior**	
Questions	Answer
Do you usually have Breakfast?	1=Never<br>2=Sometimes<br>3=Every day
Do you usually have lunch?	1=Never<br>2=Sometimes<br>3=Every day
Do you usually have dinner?	1=Never<br>2=Sometimes<br>3=Every day
Do you usually have snacks between your meals?	1=No<br>2=Yes
If yes, how many snacks do you have per day?	1=One<br>2=Two<br>3=Three or more
If yes, what type of snacks do you most usually consume?	1=Sandwich<br>2=Fruits & vegetables<br>3=Candies<br>4=Chocolate or cookies<br>5=Chips, crackers, nuts
How many times per week do you eat fast food or eat out?	1=Never<br>2=Once<br>3=Twice<br>4=Three or more
Have you ever smoked 100 cigarettes (5 packs) in your life?	1=No<br>2=Yes
During the past 30 days, on how many days did you smoke cigarettes?	1=0 days<br>2=1 or 2 days<br>3=3 to 5 days<br>4=6 to 9 days<br>5=10 to 19 days<br>6=20 to 29 days<br>7=All 30 day
During the past month, on how many days did you drink alcohol?	1=0 days<br>2=1 or 2 days<br>3=3 to 5 days<br>4=6 to 9 days<br>5=10 to 19 days<br>6=20 to 29 days<br>7=All 30 day
On an average school night, how many hours of sleep do you get?	1=4 or less hours<br>2=5 hours<br>3=6 hours<br>4=7 hours<br>5=8 hours<br>6=9 hours<br>7=10 or more hours

Supplementary Table S3: Breakfast Questions

**Breakfast Questions**	
Questions	Answer
Do you usually eat breakfast?	1=No<br>2=Yes
How many days of the week do you eat breakfast?	1=0<br>2=1<br>3=2<br>4=3<br>5=4<br>6=5<br>7=6<br>8=7
How soon after waking up do you usually have breakfast?	1=0-3 hours<br>2=> 3 hours
What do you usually eat for breakfast? You can choose more than one answer.	1=Milk<br>2=Yogurt<br>3=Cheese<br>4=Labneh<br>5=Thyme<br>6=Olives<br>7=Eggs<br>8=Cereals<br>9=Beans<br>10=Vegetables<br>11=Fruits/Fruit Juice<br>12=Bread/Baguette/Toast/Kaak<br>13=Meat (Turkey, Mortadelle)<br>14=Honey/Halawa/Jam<br>15=Manakeesh/Knefe/Croissant<br>16=Tea/Coffee/Nescafee<br><br>If other please write it down: ______________________

Supplementary Table S4: Physical Activity

**Physical Activity**		
We are interested in finding out about the kinds of physical activities that people do as part of their everyday lives. The questions will ask you about the time you spent being physically active in the last 7 days. Please answer each question even if you do not consider yourself to be an active person. Please think about the activities you do at school, as part of your house and yard work, to get from place to place, and in your spare time for recreation, exercise or sport.<br>Think about all the vigorous activities that you did in the last 7 days. Vigorous physical activities refer to activities that take hard physical effort and make you breathe much harder than normal. Think *only* about those physical activities that you did for at least 10 minutes at a time.		
	Questions	Answer
1.1	During the last 7 days, on how many days did you do vigorous physical activities like heavy lifting, digging, aerobics, or fast bicycling?	_____ days per week<br>No vigorous physical activities
If the answer is no vigorous physical activities, skip to Question 1.3		

1.2	How much time did you usually spend doing vigorous physical activities on one of those days?	_____ hours per day<br>_____ minutes per day<br>Don't Know/Not Sure
Think about all the moderate activities that you did in the last 7 days. Moderate activities refer to activities that take moderate physical effort and make you breathe somewhat harder than normal. Think only about those physical activities that you did for at least 10 minutes at a time.		
1.3	During the last 7 days, on how many days did you do moderate physical activities like carrying light loads, bicycling at a regular pace, or doubles tennis? Do not include walking.	_____ days per week<br>No moderate physical activities
If the answer is no moderate physical activities, skip to Question 1.5		
1.4	How much time did you usually spend doing moderate physical activities on one of those days?	_____ hours per day<br>_____ minutes per day<br>Don't Know/Not Sure
Think about the time you spent walking in the last 7 days. This includes at school and at home, walking to travel from place to place, and any other walking that you have done solely for recreation, sport, exercise, or leisure.		
1.5	During the last 7 days, on how many days did you walk for at least 10 minutes at a time?	_____ days per week<br>No walking
If the answer is no walking, skip to Question 1.7		
1.6	How much time did you usually spend walking on one of those days?	_____ hours per day<br>_____minutes per day<br>Don't Know/Not Sure
The last question is about the time you spent sitting on weekdays during the last 7 days. Include time spent at school, at home and during leisure time. This may include time spent sitting at a desk, visiting friends, reading, or sitting or lying down to watch television.		
1.7	During the last 7 days, how much time did you spend sitting on a week day?	_____ hours per day<br>_____ minutes per day<br>Don’t know/not sure

Supplementary Table S5: Socio-cognitive factors

**Socio-cognitive factors**	
Attitude Pro	Getting good academic grades is a good help for getting a good job
	Getting good academic grades will get me compliment from my parents
Attitude Con	Getting good academic grades means that I have to work too hard
	Getting good academic grades will cause disapproval among my friends
Social norms	My father expects me to get good academic grades
	My mother expects me to get good academic grades
	My teacher expects me to get good academic grades
Self-efficacy	I find it very easy to get good academic grades
	I find it easy to concentrate at school for getting good academic grades

	I find it easy to master the skills that are taught in class this year
	I find it easy to concentrate on school work when I am at home
	I find it easy to finish all my school work
Intention	I intend to get good academic grades

Responses: 1 = strongly disagree, 2= Disagree, 3= Neither agree nor disagree, 4= Agree 5 = strongly agree
